# Recent understanding of immunometabolic remodeling in pulmonary macrophages: homeostasis, chronic respiratory diseases, and therapeutic targeting

**DOI:** 10.3389/fphar.2026.1890878

**Published:** 2026-08-20

**Authors:** Zhenli Yuan, Ahmad Alhaskawi, Yejiang Tang, Bing Ruan

**Affiliations:** 1 Department of Thoracic Surgery, Zhuji People’s Hospital, Shaoxing, Zhejiang, China; 2 Department of Orthopedics, The First Affiliated Hospital of Zhejiang University School of Medicine, Hangzhou, China; 3 Department of Emergency, Zhuji People’s Hospital, Shaoxing, Zhejiang, China; 4 Department of Infection Disease, Zhuji People’s Hospital, Shaoxing, Zhejiang, China

**Keywords:** chronic respiratory diseases, immunometabolism, metabolic reprogramming, oxidative stress, pulmonary macrophages

## Abstract

Pulmonary macrophages are essential regulators of immune surveillance, tissue homeostasis, and inflammatory responses within the respiratory microenvironment. Emerging evidence indicates that chronic environmental stress and persistent injury induce profound immunometabolic remodeling in these cells, thereby contributing to the development and progression of chronic lung diseases. Under physiological conditions, pulmonary macrophages maintain metabolic homeostasis primarily through oxidative phosphorylation and fatty acid oxidation. However, pathological conditions drive metabolic reprogramming characterized by altered glycolysis, mitochondrial dysfunction, oxidative stress, lipid dysregulation, and disturbed iron homeostasis, leading to persistent inflammation, impaired tissue repair, and progressive remodeling. Recent studies have further highlighted the critical interplay between metabolic pathways, redox signaling, and immune regulation in shaping macrophage phenotypes and functions. Importantly, therapeutic strategies targeting macrophage metabolism and redox balance, together with advances in macrophage-directed drug delivery systems, have emerged as promising approaches for modulating pulmonary inflammation and tissue injury. This review summarizes recent understanding of immunometabolic remodeling in pulmonary macrophages under homeostatic and pathological conditions and discusses emerging therapeutic perspectives targeting macrophage metabolism in chronic respiratory diseases.

## 1 Introduction

**FIGURE 1:**
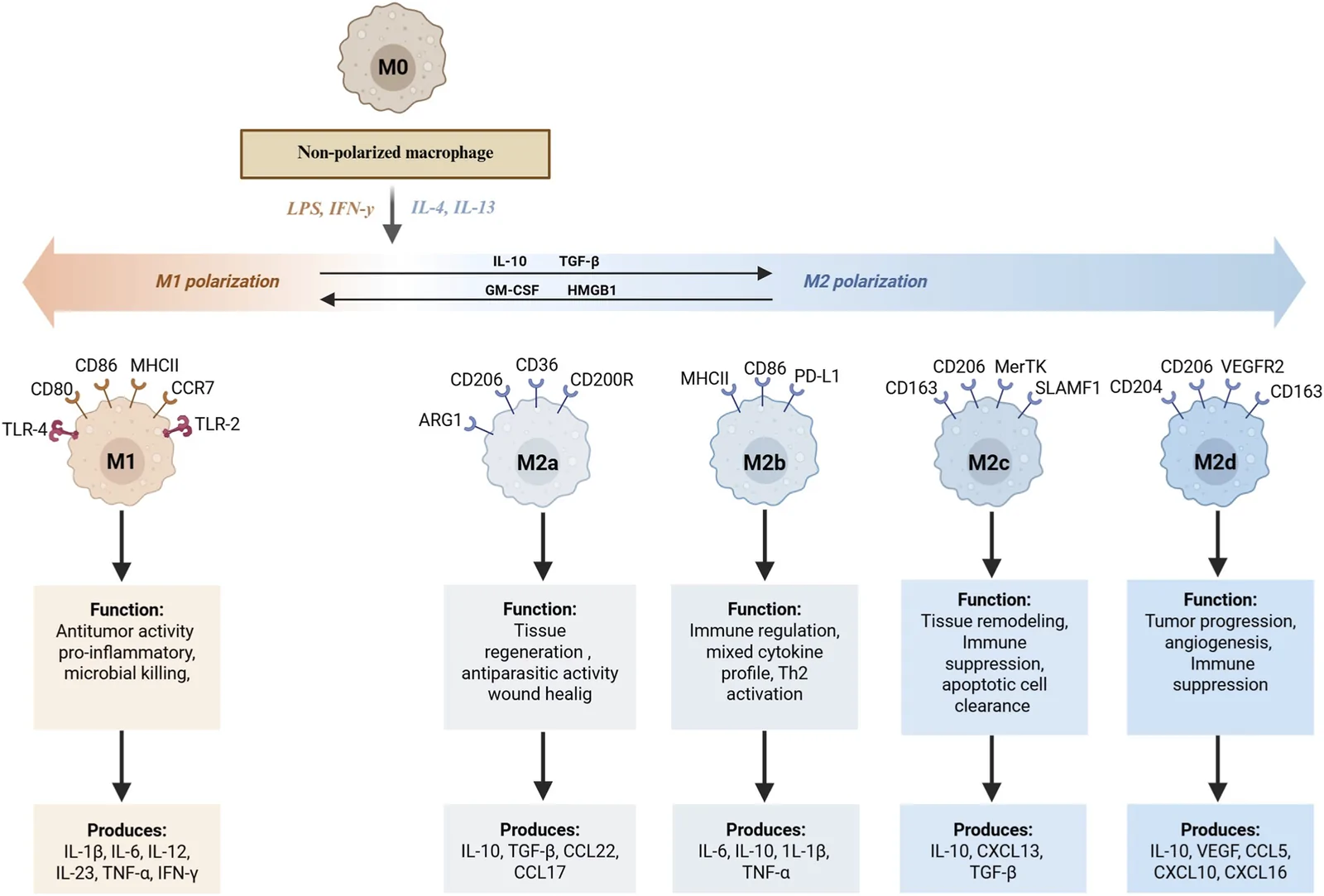
Overview of macrophage polarization pathways, highlighting the phenotypic markers, biological functions, and cytokine profiles of M1 and M2 macrophage subsets.

The lung is a highly specialized organ that maintains a delicate balance between efficient gas exchange and continuous exposure to environmental challenges, including pathogens, allergens, and particulate matter (Pouptsis et al., 2025). Central to this balance are pulmonary macrophages, a heterogeneous population of innate immune cells that orchestrate immune surveillance, tissue homeostasis, and inflammatory responses. These cells serve as the first line of defense against inhaled insults and play critical roles in shaping the local microenvironment through dynamic interactions with epithelial, endothelial, and stromal compartments (Melo et al., 2021; Aegerter et al., 2022). Pulmonary macrophages comprise distinct subsets with unique developmental origins and functional specializations, primarily including alveolar macrophages (AMs) and interstitial macrophages (IMs). These populations exhibit remarkable plasticity, enabling them to respond appropriately to physiological and pathological stimuli. However, under conditions of chronic stress or persistent injury, this adaptive capacity may become dysregulated. Aberrant immunometabolic reprogramming has been increasingly implicated in the pathogenesis of a wide spectrum of chronic lung diseases (Pervizaj-Oruqaj et al., 2024; Hou et al., 2021). Disease-associated macrophages frequently exhibit distinct metabolic programs that contribute to pathological inflammation, tissue remodeling, and immune dysregulation. Using the conventional M1/M2 framework as a broad conceptual model, M1-like macrophages are generally associated with enhanced glycolytic flux, supporting pro-inflammatory cytokine production and antimicrobial activity, whereas M2-like macrophages more commonly rely on oxidative phosphorylation and lipid metabolism, functions linked to tissue repair, fibrosis, and immune regulation (Mitsui and Satoh, 2025; Ni et al., 2023; Gupta and Sarangi, 2023; Strizova et al., 2023; Fujisaka et al., 2016) (Figure 1). However, this framework represents a simplified experimental model rather than a set of fixed or mutually exclusive cell states (Murray et al., 2014; Xue et al., 2014). This limitation is particularly relevant in the lung, where resident AMs, IMs, and recruited monocyte-derived macrophages occupy distinct anatomical niches and acquire context-dependent transcriptional and metabolic programs during inflammation and tissue remodeling (Aegerter et al., 2022). Consistent with this complexity, single-cell transcriptomic studies have identified disease-associated macrophage populations, including SPP1-, TREM2-, and CD9-expressing subsets, that extend beyond the conventional M1/M2 classification (Fabre et al., 2023). Moreover, emerging evidence highlights the interplay between metabolic intermediates and signaling pathways, such as hypoxia-inducible factors (HIFs), AMP-activated protein kinase (AMPK), and mechanistic target of rapamycin (mTOR), in regulating macrophage function within the pulmonary microenvironment (Mu et al., 2025; Lee et al., 2015; Bao J. et al., 2025). These findings underscore the central role of immunometabolic regulation in pulmonary macrophage biology and provide a strong rationale for targeting macrophage metabolism as a potential therapeutic strategy. Interventions aimed at modulating redox balance, metabolic flux, and intracellular signaling networks, including NRF2 activators, metabolic enzyme inhibitors, and pathway-specific modulators, have shown therapeutic potential (Checa and Aran, 2020; Duran et al., 2016). In parallel, advances in targeted drug delivery systems offer new opportunities to selectively manipulate macrophage function within the lung, thereby enhancing therapeutic efficacy while minimizing systemic toxicity. In this review, we provide a comprehensive overview of the immunometabolic regulation of pulmonary macrophages, beginning with their heterogeneity and metabolic characteristics under homeostatic conditions. We then examine the role of macrophage immunometabolism across chronic lung diseases, highlighting shared and disease-specific mechanisms. Finally, we discuss emerging therapeutic strategies targeting macrophage metabolism.

## 2 Pulmonary macrophages

**FIGURE 2:**
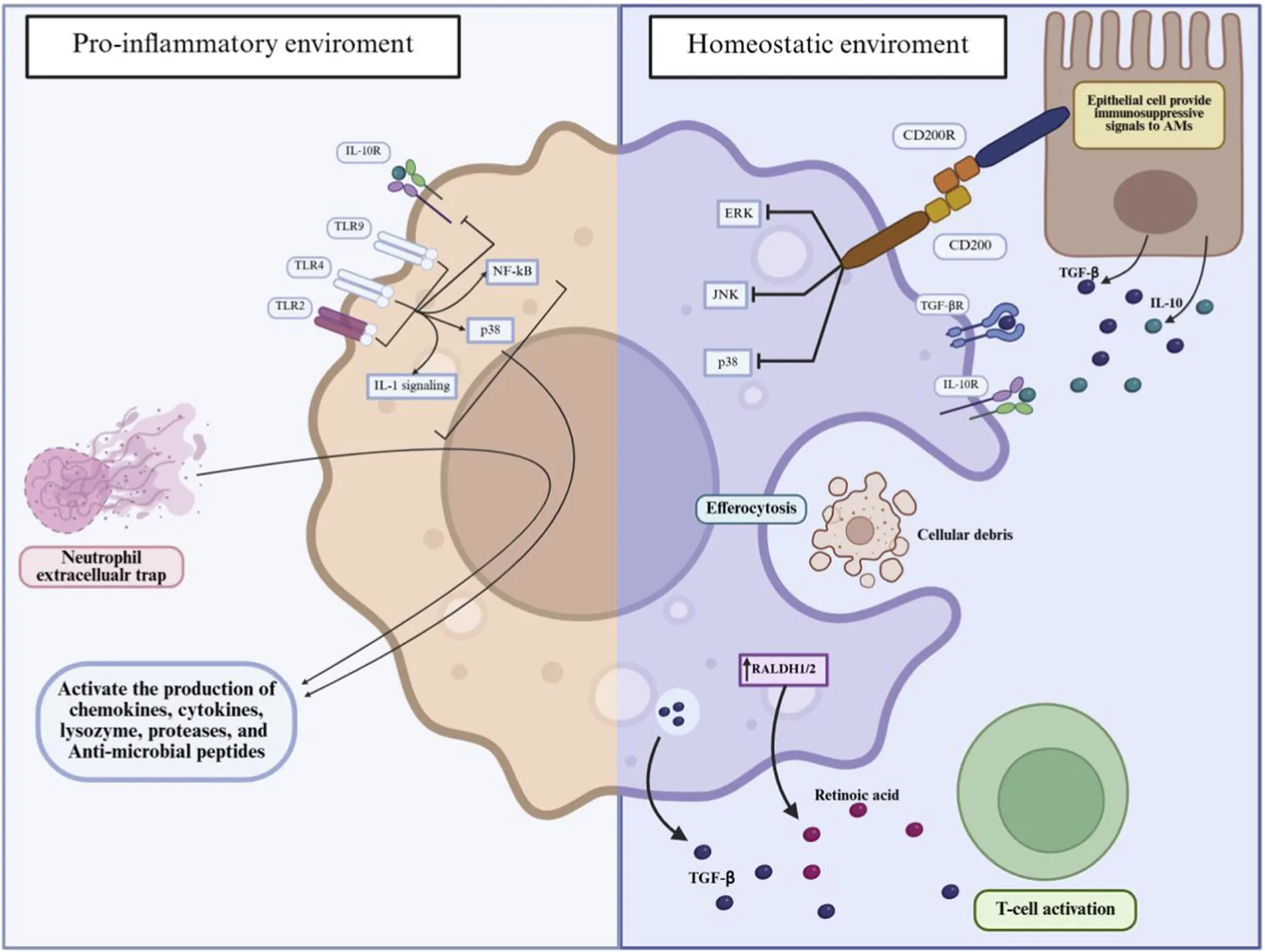
Alveolar macrophage functions under pro-inflammatory and homeostatic conditions, highlighting inflammatory signaling, cytokine production, efferocytosis, epithelial-macrophage crosstalk, and maintenance of pulmonary immune homeostasis.

The pulmonary macrophage compartment is composed of phenotypically and functionally distinct populations that are spatially organized and adapted to specific microenvironmental niches. At steady state, lung-resident macrophages are classified into alveolar macrophages (AMs) and interstitial macrophages (IMs), each exhibiting unique developmental origins, transcriptional programs, and metabolic characteristics that support tissue homeostasis (Aegerter et al., 2022; Jin et al., 2026). AMs primarily originate from fetal monocyte precursors and establish long-lived, self-renewing populations within the alveolar lumen. Their maintenance is largely independent of circulating monocytes under homeostatic conditions and is critically regulated by local factors such as transforming growth factor-β (TGF-β) and granulocyte-macrophage colony-stimulating factor (GM-CSF), which drive lineage-specific transcriptional programs. AMs are primarily responsible for maintaining alveolar sterility and surfactant homeostasis (Guilliams et al., 2013; Hussell and Bell, 2014). They exhibit a tolerogenic phenotype, limiting excessive inflammatory responses to innocuous inhaled particles while retaining the capacity for rapid activation upon pathogenic challenge. This balanced responsiveness is achieved through tight regulation of pattern recognition receptor signaling and anti-inflammatory mediators (Allard et al., 2019; Roquilly et al., 2020a; Roquilly et al., 2020b) (Figure 2). Normally, pulmonary macrophages exhibit a metabolic profile optimized for long-term maintenance and low-grade immune surveillance. AMs rely predominantly on oxidative phosphorylation (OXPHOS) and lipid metabolism to sustain their homeostatic functions within the surfactant-rich alveolar niche. This metabolic profile reflects their continuous exposure to and processing of surfactant-derived lipids rather than generalized nutrient limitation, as the lung parenchyma is highly vascularized and normally receives abundant oxygen and circulating nutrients. In addition to lipid metabolism, mitochondrial integrity and redox balance are essential for maintaining macrophage quiescence. Basal levels of reactive oxygen species (ROS) are tightly regulated through antioxidant systems, including NRF2-mediated pathways, preventing inappropriate inflammatory activation while preserving antimicrobial capacity (Willinger, 2026; Malla et al., 2024). In contrast, IMs represent a more heterogeneous and dynamically replenished population residing within the lung parenchyma, with contributions from both embryonic progenitors and bone marrow-derived monocytes. IMs are closely associated with immune modulation within the lung tissue. They contribute to antigen presentation, regulation of adaptive immune responses, and maintenance of tissue integrity through interactions with fibroblasts, endothelial cells, and infiltrating leukocytes (Peng et al., 2025; Schyns et al., 2019). The functional plasticity of these macrophage populations enables rapid adaptation to environmental fluctuations, including changes in oxygen tension, nutrient availability, and cellular stress signals (Chakarov et al., 2019; Schyns et al., 2018). IMs display greater metabolic heterogeneity, reflecting their diverse functional roles and microenvironmental contexts. While oxidative metabolism remains dominant under homeostatic conditions, these cells retain the capacity to rapidly shift toward glycolytic pathways when required, enabling swift responses to tissue perturbation (Li et al., 2026; Ogger and Byrne, 2021).

The metabolic programming of lung macrophages is inseparable from their local microenvironment. Factors such as oxygen gradients, mechanical forces, and the biochemical composition of alveolar lining fluid collectively shape macrophage metabolism and function (Ogger and Byrne, 2021; Guo et al., 2022). For example, the relatively high oxygen availability in the alveolar space supports mitochondrial respiration, whereas localized hypoxic niches within the interstitium may influence metabolic flexibility and signaling pathways (Garcia et al., 2021; Schumacker, 2011). Furthermore, epithelial-macrophage crosstalk plays a critical role in sustaining macrophage identity and metabolic homeostasis. Epithelial-derived signals, including GM-CSF, not only regulate macrophage differentiation but also modulate key metabolic pathways that maintain cellular fitness and immune balance (Zhou et al., 2024; Lagowala et al., 2024).

## 3 Macrophage immunometabolism in chronic lung diseases

### 3.1 Asthma

**FIGURE 3:**
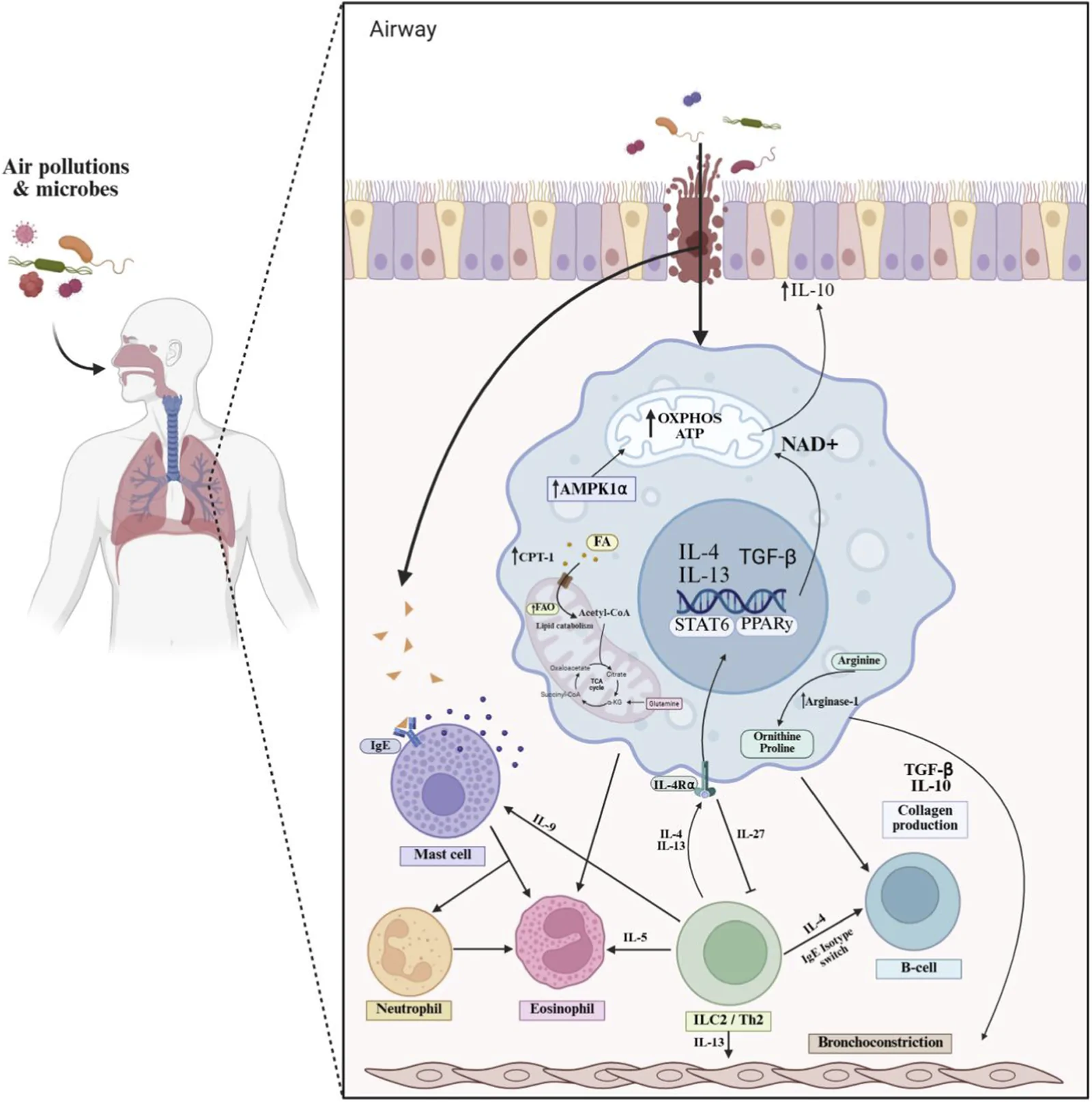
Air pollutants and microbial stimuli induce immunometabolic reprogramming of pulmonary macrophages, promoting IL-4/IL-13-mediated activation, enhanced OXPHOS and FAO, and cytokine-driven crosstalk with immune cells, thereby contributing to airway inflammation, remodeling, and bronchoconstriction.

Asthma is a heterogeneous chronic inflammatory airway disorder characterized by complex interactions between immune dysregulation, epithelial dysfunction, and structural remodeling (Fuhlbrigge and Sharma, 2025). While traditionally viewed through the lens of adaptive type 2 immunity, it is now increasingly recognized that pulmonary macrophages serve as important regulators of disease initiation, amplification, and resolution failure (Fuhlbrigge and Sharma, 2025; Lambrecht et al., 2025). In asthmatic airways, macrophages integrate environmental exposures, including allergens, pollutants, and microbial products, with intrinsic metabolic programs, thereby translating external cues into context-dependent inflammatory outputs. This integrative role is particularly evident in the setting of chronic disease, where macrophage phenotypes are shaped not only by cytokine signaling but also by sustained alterations in cellular metabolism, redox balance, and tissue microenvironmental constraints (van der Veen et al., 2020; Han et al., 2023) (Table 1). In allergic asthma, macrophages are predominantly polarized toward the alternatively activated M2-like phenotype under the influence of IL-4 and IL-13, leading to enhanced Th2 immune responses, eosinophilic infiltration, mucus production, and airway hyper-responsiveness (Saradna et al., 2018) (Figure 3). These macrophages contribute to airway remodeling through secretion of TGF-β, fibronectin, and matrix metalloproteinases, while simultaneously producing chemokines such as CCL17 and CCL22 that sustain type 2 immune cell recruitment (Britt et al., 2023). A study found that HDAC10 plays a critical epigenetic role in allergic asthma by promoting macrophage M2 polarization through STAT3 deacetylation. Mechanistically, HDAC10 directly interacted with STAT3, enhanced PI3K/Akt-STAT3 signaling, and subsequently increased the expression of M2-associated markers such as Arg1, Fizz1, and Ym1, thereby aggravating airway inflammation and remodeling in asthma models (Zhong et al., 2023). Lechner et al. reported that repeated house dust mite allergen exposure induces a TNF-dependent inflammatory memory in macrophages, resulting in persistent metabolic and epigenetic reprogramming with enhanced production of CCL17, cysteinyl leukotrienes, IL-6, and PGE2 in allergic asthma. Their study further identified the FPR2-TNF-2-hydroxyglutarate-PGE2/EP2 signaling axis and KDM1A-mediated histone remodeling as key mechanisms underlying trained macrophage immunity that may contribute to chronic type 2 airway inflammation and asthma exacerbation (Lechner et al., 2022). Furthermore, Wan et al. revealed that monocytes/macrophages, particularly AMs, are the predominant inflammatory cell population in asthma-COPD overlap (ACO) and exhibit a reduced senescence phenotype characterized by lower SenMayo enrichment scores and decreased CDKN1A expression. Their study further identified PPARγ as a key regulator of AMs senescence and suggested that altered senescence-associated signaling in these macrophages may contribute to chronic airway inflammation and disease severity in ACO (Wan et al., 2024). Bao et al. Found that polyvinyl chloride (PVC) nanoplastics aggravate allergic asthma by accumulating in AMs and inducing R-loop formation through RNASEH1 suppression, which subsequently activates the cGAS-STING inflammatory pathway. Their findings further showed that STING activation promoted macrophage-driven airway inflammation, characterized by enhanced Th2 cytokine production, inflammatory cell infiltration, airway hyperresponsiveness, and pulmonary remodeling in asthmatic mice (Bao Q. et al., 2025).

**TABLE 1:** Asthma-associated immunometabolic alterations in alveolar macrophages and their functional relevance.

Metabolic or biochemical finding	Associated functional consequence	References
Elevated ROS levels	Pulmonary injury and increased TNF-α/IL-1β production	Park et al. (2009), Calhoun and Bush (1990)
Increased HO-1 expression	Associated with oxidant stress and increased heme degradation/CO production	Harju et al. (2002a)
Elevated leukotriene B4/E4 production	Bronchoconstriction and airway hyperresponsiveness	Arm et al. (1989), Chavis et al. (1991)
Reduced 5-LOX expression and activity following HDM exposure	Decreased 5-HETE and LTB_4_ production with enhanced prostanoid synthesis	Henkel et al. (2019)
Enhanced CPT-associated FAO signaling	Increased FAO metabolism	Al-Khami et al. (2017)
Increased Arg1 with reduced NOS2 expression	Enhanced ornithine and proline biosynthesis	Chang et al. (2000), Vercelli (2003)

In contrast, non-type 2 or severe asthma phenotypes are often characterized by macrophages with a predominantly pro-inflammatory profile, often associated with neutrophilic infiltration and corticosteroid resistance. These cells exhibit increased production of IL-1β, IL-6, and TNF-α, alongside enhanced inflammasome activation, highlighting a shift toward innate immune driven pathology (Liu T. et al., 2024; Lee et al., 2025). Importantly, these phenotypic states are not fixed but exist along a continuum, with macrophages dynamically transitioning between activation states depending on the evolving inflammatory and metabolic milieu. Han et al. identified a protective CD39^+^CD9^+^ IM subset that was reduced during neutrophilic asthma and restored by IL-23 blockade. These cells suppressed Th17-driven inflammation by attaching to neutrophils through CD9 and degrading extracellular ATP through CD39, thereby limiting NETosis and airway inflammation (Han et al., 2024a). Moreover, Matsuda et al. demonstrated that CCR5^+^ interstitial macrophages were increased in steroid-resistant severe asthma and contributed to subepithelial fibrosis through TGF-β production. Pharmacological CCR5 blockade with maraviroc reduced TGF-β–producing interstitial macrophages and attenuated airway fibrosis without substantially affecting eosinophil, Th2-cell, ILC2, neutrophil, or total macrophage accumulation (Matsuda et al., 2024).

In asthmatic inflammation, pro-inflammatory macrophages undergo a shift toward aerobic glycolysis, characterized by increased glucose uptake, enhanced activity of key glycolytic enzymes, and accumulation of intermediate metabolites. This metabolic configuration supports rapid ATP production and provides biosynthetic precursors for cytokine synthesis (Lv et al., 2025; Albers et al., 2025). Beyond energy generation, glycolytic intermediates act as signaling molecules; for example, succinate accumulation stabilizes HIF-1α, promoting transcription of IL-1β and other pro-inflammatory genes (Suresh et al., 2023). Concurrently, disruption of the tricarboxylic acid (TCA) cycle leads to the generation of metabolites such as itaconate, which can exert immunoregulatory effects, reflecting the dual nature of metabolic pathways in shaping inflammatory responses. These alterations are particularly relevant in the hypoxic microenvironments that develop within inflamed airway tissues, where oxygen limitation further reinforces glycolytic dependency and HIF-mediated signaling (Albers et al., 2026). In parallel, macrophages involved in tissue remodeling and resolution exhibit a metabolic profile dominated by oxidative phosphorylation (OXPHOS) and fatty acid oxidation (FAO). In asthma, type 2 cytokine signaling enhances mitochondrial biogenesis and promotes lipid utilization, supporting alternative activation programs associated with tissue repair (Qian et al., 2022; Lambrecht et al., 2019). However, sustained activation of these pathways contributes to pathological remodeling, including subepithelial fibrosis and airway wall thickening. Mitochondrial function in these macrophages is tightly linked to ROS production, which at physiological levels serves as a signaling mechanism but, when dysregulated, drives oxidative stress and cellular dysfunction. The balance between mitochondrial respiration and ROS generation is therefore a critical determinant of macrophage behavior in the asthmatic lung (Zhang et al., 2024; Weinberg and Chandel, 2025). Furthermore, altered lipid mediator profiles in the asthmatic airways leads to increased production of pro-inflammatory eicosanoids such as leukotrienes and prostaglandins. Macrophages play a central role in the synthesis and metabolism of these mediators, and dysregulation of lipid handling can profoundly affect inflammatory outcomes (Sanak, 2016). In addition, ROS-mediated activation of NF-κB and MAPK pathways enhances inflammatory gene expression, while oxidative modification of metabolic enzymes can further disrupt cellular metabolism (Michaeloudes et al., 2022; Yuan et al., 2025). At the same time, sustained allergen exposure and inflammatory-cell activation generate an excessive oxidative burden that may compromise NRF2-dependent antioxidant responses in pulmonary macrophages. In patients with atopic asthma, segmental allergen challenge reduced Nrf2 protein expression and DNA-binding activity in alveolar macrophages, accompanied by decreased expression of the NRF2-regulated antioxidant enzyme superoxide dismutase 1 (Dworski et al., 2011). The partial preservation of these responses following vitamin E supplementation suggests that allergen-induced oxidative stress contributes to Nrf2 suppression. Consequently, insufficient activation of antioxidant and cytoprotective pathways may permit persistent ROS accumulation and sustained activation of NF-κB- and MAPK-dependent inflammatory signaling (Dworski et al., 2011). This interaction between impaired antioxidant defense and metabolic reprogramming may establish a self-reinforcing cycle that perpetuates airway inflammation and tissue injury (Li et al., 2013). Nevertheless, the upstream mechanisms responsible for NRF2 impairment and their potential variation across asthma endotypes remain incompletely understood. In addition, metabolic–epigenetic crosstalk may contribute to the persistence of macrophage functional states, as metabolites such as acetyl-CoA and α-ketoglutarate regulate histone modifications and gene-expression programs (Sharma et al., 2022).

### 3.2 Chronic obstructive pulmonary disease

Chronic obstructive pulmonary disease (COPD) is a progressive disorder characterized by persistent airflow limitation, emphysematous destruction, and chronic bronchitis, driven predominantly by long-term exposure to noxious particles such as cigarette smoke and biomass fuels (Christenson et al., 2022). In this setting, pulmonary macrophages accumulate in large numbers within the airway lumen, interstitium, and alveolar spaces, where they adopt a disease-specific phenotype that differs fundamentally from the macrophage programs observed in asthma (Christenson et al., 2022; Li X. et al., 2025). Rather than a cytokine-dominated polarization alone, COPD macrophages are defined by chronic oxidative stress, mitochondrial dysfunction, impaired proteostasis, and altered iron and lipid handling, all of which converge on a distinct immunometabolic state that sustains tissue injury and limits effective resolution (Qin et al., 2025) (Table 2). A hallmark of COPD AMs is mitochondrial remodeling and bioenergetic insufficiency. Chronic exposure to cigarette smoke induces structural and functional damage to mitochondria, including loss of membrane potential, impaired electron transport chain activity, and accumulation of mitochondrial DNA damage. These alterations result in inefficient oxidative phosphorylation and increased electron leakage, leading to excessive generation of mitochondrial reactive oxygen species (mtROS) (Kanithi et al., 2022; Liang L. et al., 2025). Unlike the regulated metabolic shifts observed in other inflammatory contexts, mitochondrial dysfunction in COPD represents a maladaptive and persistent defect, which not only amplifies inflammatory signaling but also compromises cellular fitness. Defective mitophagy further exacerbates this process, allowing damaged mitochondria to accumulate and perpetuate a cycle of oxidative stress and inflammatory activation (Tulen et al., 2024). Furthermore, COPD macrophages exhibit metabolic inflexibility, characterized by an inability to appropriately switch between glycolytic and oxidative pathways in response to environmental cues. While increased glycolytic activity has been reported in response to acute inflammatory stimuli, chronic smoke exposure often leads to an overall decline in metabolic efficiency, with reduced ATP generation and impaired biosynthetic capacity. This dysfunctional metabolic state is closely linked to impaired phagocytosis and defective efferocytosis, two critical functions required for the clearance of pathogens and apoptotic cells. As a result, AMs in COPD fail to effectively resolve inflammation, contributing to the persistence of airway injury and recurrent exacerbations (Qin et al., 2025; Zhou et al., 2025). Another defining feature of COPD macrophage biology is dysregulated iron metabolism. Repeated exposure to particulate matter and cigarette smoke leads to accumulation of iron within AMs, partly due to increased uptake and impaired export mechanisms. Elevated intracellular iron levels promote the generation of highly reactive hydroxyl radicals through Fenton chemistry, thereby intensifying oxidative stress and lipid peroxidation (Ghio et al., 2008; Cloonan et al., 2017). Iron-loaded macrophages display a pro-inflammatory phenotype and reduced antimicrobial capacity, creating a permissive environment for chronic bacterial colonization, which is a common feature of advanced COPD. This disruption of iron homeostasis also intersects with mitochondrial dysfunction, further aggravating cellular damage and metabolic dysregulation (Ho et al., 2022; Zhang W. Z. et al., 2020). Cloonan et al. demonstrated that iron-responsive element-binding protein 2 (IRP2) promotes cigarette smoke-induced COPD by driving mitochondrial iron overload, mitochondrial dysfunction, pulmonary inflammation, and emphysematous lung injury. IRP2 deficiency protected mice from bronchitis and emphysema, whereas mitochondrial iron chelation markedly alleviated both early and established disease (Cloonan et al., 2016). Nevertheless, lipid metabolism is profoundly dysregulated in COPD, with AMs exhibiting features of lipid overload, defective lipid handling, and mitochondrial metabolic dysfunction. Macrophages accumulate oxidized lipids and cholesterol, promoting foam cell-like phenotypes similar to those observed in atherosclerosis. These lipid-laden macrophages display impaired efferocytosis together with increased secretion of proteases and inflammatory mediators, thereby contributing to alveolar destruction and emphysema progression (Fujii et al., 2021; Liang Q. et al., 2025).

**TABLE 2:** Immunometabolic alterations in alveolar macrophages in COPD and their associated cellular and pathological consequences.

Immunometabolic alteration	Associated cellular or pathological consequence	References
Reduced mROS response following stimulation	Defective bacterial clearance	Bewley et al. (2017)
Increased inducible iNOS synthase expression	Excess nitric oxide generation	Ichinose et al. (2000)
Elevated production of ROS, mROS, and superoxide	Enhanced oxidative stress	Bewley et al. (2017), Rahman and MacNee (1996), Xia et al. (2007)
Decreased glutamyl-cysteine ligase activity	Impaired glutathione biosynthesis	Harju et al. (2002b)
Insufficient compensatory glycolytic adaptation	Failure to meet cellular energy requirements	O'Beirne et al. (2020)
Reduced mitochondrial membrane potential	Impaired phagocytic capacity	Belchamber et al. (2019)
Reduced mitochondrial coupling efficiency and oxidative phosphorylation	Metabolic dysfunction and altered macrophage phenotype	O'Beirne et al. (2020)
Enhanced intracellular iron retention	Increased ROS-mediated injury	(Philippot et al. (2014)
Increased proton leakage across mitochondria	Mitochondrial inefficiency	O'Beirne et al. (2020)
Upregulated HIF-1α signaling	Enhanced glycolytic activity	Russell et al. (2016)
Elevated A2BR expression	Dysregulated adenosine metabolism	Zhou et al. (2010)

Mitochondria play an important role in lipid metabolism through regulation of fatty acid synthesis (FAS) and FAO. Altered mitochondrial fatty acid metabolism has been increasingly linked to macrophage polarization and immune dysfunction in COPD (Wedan et al., 2024; Ran et al., 2026). IL-4-driven activation of SREBP1 enhances *de novo* lipogenesis and ROS production, promoting M2-associated metabolic reprogramming, whereas the mitochondrial ROS-Fgr kinase axis has been implicated in pro-inflammatory M1 activation and impaired FAO (Bidault et al., 2021; Plantier et al., 2012). In parallel, disrupted synthesis of lipid mediators further shifts macrophages toward a persistent inflammatory state by enhancing pro-inflammatory signaling while reducing the production of pro-resolving mediators required for tissue repair and inflammation resolution (Chen et al., 2019). In addition, sphingolipid metabolism contributes to COPD pathogenesis. Excessive ceramide accumulation impairs macrophage phagocytosis through suppression of Rac1 signaling, disrupts cytoskeletal organization, and reduces apoptotic cell clearance, thereby exacerbating emphysematous injury (Koike et al., 2018). Its downstream metabolite sphingosine-1-phosphate (S1P) may also participate in these processes, suggesting that targeting mitochondrial and sphingolipid metabolic pathways could represent a promising strategy for restoring macrophage function in COPD (Petrusca et al., 2010).

At the signaling level, COPD macrophages are characterized by sustained activation of stress-responsive pathways, including NF-κB, p38 MAPK, and inflammasome-associated signaling cascades (Alanazi et al., 2025; Ahmadi et al., 2023; Zaynagetdinov et al., 2016). These pathways are closely intertwined with metabolic dysfunction and are further amplified by oxidative stress. In contrast to other disease contexts, the antioxidant response in COPD is often insufficient, with impaired activation of NRF2-dependent pathways contributing to an inability to counterbalance oxidative injury (Gao et al., 2025; Fan et al., 2023). Additionally, dysregulation of metabolic sensors such as AMPK has been implicated in reduced mitochondrial biogenesis and impaired cellular homeostasis, further reinforcing the dysfunctional macrophage phenotype (He et al., 2025; Balnis et al., 2020). Abnormalities in nitrogen metabolism are also closely linked to COPD pathogenesis. Increased inducible nitric oxide synthase (iNOS) expression in macrophages and inflammatory cells enhances nitric oxide (NO) production, which reacts with superoxide to generate reactive nitrogen species such as peroxynitrite (Seimetz et al., 2011; Ichinose et al., 2000). Excessive RNS formation induces oxidative and nitrosative stress, promotes protein nitration, mitochondrial dysfunction, and amplifies chronic airway inflammation. Nitrotyrosine accumulation, a marker of RNS-mediated injury, is significantly elevated in COPD airways and correlates with airflow limitation severity (Ichinose et al., 2000; Bhowmik et al., 2005). Functionally, the immunometabolic abnormalities translate into key pathological features of COPD. Macrophages contribute to chronic inflammation through sustained cytokine and chemokine production, promote tissue destruction via release of matrix-degrading enzymes such as matrix metalloproteinases, and impair host defense through defective phagocytic activity (Watanabe et al., 2019). Importantly, their inability to efficiently clear apoptotic cells leads to secondary necrosis and release of damage-associated molecular patterns, which further perpetuate inflammation. The cumulative effect of these processes is a self-sustaining cycle of injury, inflammation, and defective repair that underlies disease progression.

### 3.3 Idiopathic pulmonary fibrosis

**FIGURE 4:**
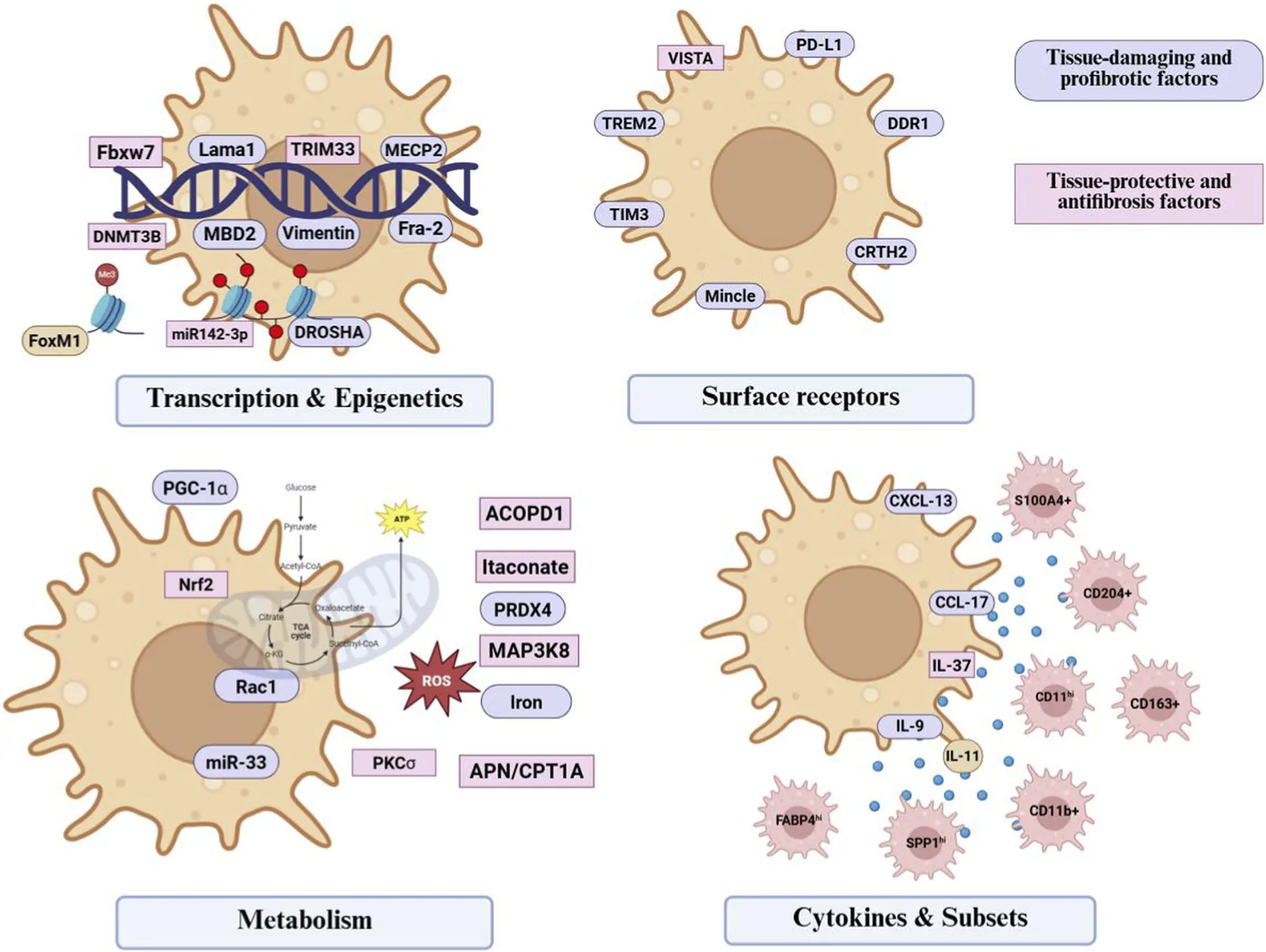
Key mechanisms underlying pulmonary macrophage dysregulation in fibrotic lung disease, including transcriptional and epigenetic regulation, surface receptor signaling, metabolic reprogramming, and cytokine/subset heterogeneity. Pink labels indicate tissue-protective and antifibrotic factors, whereas purple labels represent tissue-damaging and profibrotic mediators.

Idiopathic pulmonary fibrosis (IPF) is a progressive interstitial lung disease characterized by irreversible scarring, architectural distortion, and declining pulmonary function (Richeldi et al., 2017). In contrast to inflammatory airway diseases, IPF is primarily driven by aberrant wound healing and persistent fibrogenesis, in which pulmonary macrophages act as critical regulators of epithelial injury responses, fibroblast activation, and ECM deposition (Lu et al., 2025; Zabihi et al., 2026) (Figure 4). Rather than mounting a classical inflammatory program, macrophages in IPF acquire a profibrotic, metabolically specialized phenotype that sustains tissue remodeling and disrupts normal repair processes (Table 3). A defining feature of IPF is the expansion of monocyte-derived macrophages within fibrotic niches (Mao et al., 2026). Single-cell transcriptomic analyses have identified distinct macrophage subsets enriched in fibrotic lungs, characterized by expression of markers such as SPP1 (osteopontin), TREM2, and CD163 (Cruz Tleugabulova et al., 2024; Ding et al., 2025). These monocyte-derived macrophages localize to areas of active fibrosis, including fibroblastic foci, where they engage in direct crosstalk with fibroblasts and epithelial cells. Functionally, they secrete a range of profibrotic mediators, including TGF-β, platelet-derived growth factor (PDGF), and connective tissue growth factor (CTGF), which collectively promote fibroblast proliferation, differentiation into myofibroblasts, and excessive ECM accumulation (Behmoaras et al., 2025; Antoniades et al., 1990; Effendi and Nagano, 2022). Fibrotic lung macrophages exhibited upregulated glycolytic mediators, including Glut-1, HK2, PFKFB3, and LDHB, together with increased glucose consumption, indicating profound metabolic reprogramming. Importantly, glycolytic inhibition reversed the pro-fibrotic macrophage phenotype, highlighting Glut-1-associated glycolysis as a critical mechanism in pulmonary fibrosis progression (El-Chemaly et al., 2013; Xie et al., 2017; Yin et al., 2019; Xu et al., 2021). Furthermore, TGF-β signaling, a central driver of fibrosis, directly influences macrophages metabolism by promoting mitochondrial biogenesis and lipid utilization. This metabolic state not only facilitates energy production but also generates intermediates required for collagen synthesis and ECM remodeling (Hu et al., 2018; Budi et al., 2021; Bueno et al., 2020). Mitochondrial dysfunction and excessive ROS production in fibrotic macrophages may promote a profibrotic phenotype by enhancing TGF-β/SMAD signaling and the release of mediators that stimulate fibroblast activation, myofibroblast differentiation, and extracellular matrix deposition (Szabo et al., 2026; Cloonan, 2017; Chen et al., 2025; Gauldie et al., 2006). Moreover, alterations in the ACOD1/itaconate metabolic axis represent an important feature of macrophage dysfunction in IPF. Normally, itaconate produced by airway macrophages acts as an immunometabolic regulator that restrains excessive inflammatory and fibrotic responses through modulation of mitochondrial metabolism and oxidative stress pathways (Ogger et al., 2020; Michalaki et al., 2024). However, patients with IPF exhibit reduced ACOD1 expression in airway macrophages and decreased itaconate levels in bronchoalveolar lavage fluid. Impaired ACOD1-itaconate signaling promotes a profibrotic macrophage phenotype associated with enhanced fibroblast activation and ECM deposition. Experimental restoration of itaconate signaling attenuated pulmonary fibrosis, highlighting the macrophage ACOD1-itaconate pathway as a potential therapeutic target in IPF (Ogger et al., 2020; Michalaki et al., 2024). In addition, IPF lung macrophages exhibit increased mitochondrial calcium uniporter (MCU) expression, mitochondrial calcium accumulation, and enhanced fatty acid oxidation (Gu et al., 2019). MCU-mediated mitochondrial ROS production activated the PGC-1α/FAO pathway, promoting profibrotic macrophage polarization and fibrosis progression. Inhibition of MCU or suppression of fatty acid oxidation reversed macrophage profibrotic activation and markedly attenuated pulmonary fibrosis (Gu et al., 2019).

**TABLE 3:** Targeting therapy for macrophage-associated iron dysregulation in idiopathic pulmonary fibrosis.

Therapeutic strategy	Representative agent/Method	Proposed mechanism and therapeutic role
Intracellular iron reduction	Deferoxamine (DFO)	Functions as an iron chelator that lowers macrophage iron accumulation and suppresses profibrotic M2-associated markers such as CD206 and ARG1 (Park et al., 2021)
Regulation of iron export	Hepcidin-based modulation	Promotes ferroportin (SLC40A1)-mediated iron efflux, thereby reducing cellular iron overload (Xie et al., 2025)
Heme metabolism targeting	HO-1 modulation	Regulates heme degradation and macrophage polarization through control of carbon monoxide, biliverdin, and iron metabolism (Ye et al., 2008)
Ferroptosis suppression	Ferrostatin-1 (Fer-1)	Limits ferroptotic cell death and attenuates inflammation- and fibrosis-associated lung injury (Aerbajinai et al., 2019)
Advanced delivery technologies	Nanoparticle-based or inhalable formulations	Improves pulmonary targeting efficiency while minimizing systemic exposure and adverse effects (Han et al., 2024b)
Combination therapeutic approaches	DFO combined with esomeprazole	Delays fibrotic progression by modulating iron homeostasis and macrophage–fibroblast interactions (Cheng et al., 2025)

Disrupted iron handling in macrophages represents another major immunometabolic feature of IPF. Similar to observations in COPD, fibrotic lungs often contain hemosiderin-rich AMs, reflecting persistent microvascular leakage or excessive iron uptake (Kono et al., 2024). These iron-overloaded macrophages contribute to fibrosis through multiple mechanisms, including enhanced ROS generation, secretion of profibrotic cytokines, and stimulation of fibroblast proliferation and myofibroblast activation (Li Y. et al., 2024). Notably, reduced expression of the transferrin receptor CD71 on AMs in IPF may represent an adaptive response to intracellular iron excess, ultimately leading to accumulation of extracellular transferrin-bound iron within the fibrotic microenvironment (Allden et al., 2019; Byrne et al., 2019). Excess iron further amplifies oxidative stress through Fenton chemistry, producing highly reactive hydroxyl radicals that aggravate epithelial injury and tissue remodeling. Iron accumulation also promotes macrophage polarization toward a profibrotic phenotype and has been increasingly linked to ferroptosis-related lung injury in advanced fibrosis (Hu et al., 2024). Elevated iron deposition and lipid peroxidation within fibrotic lungs may trigger ferroptotic death of alveolar epithelial cells and macrophages, thereby releasing damage-associated molecular patterns (DAMPs) that further activate fibroblasts and perpetuate fibrogenic signaling (Kim et al., 2010; Yao et al., 2025). Experimental studies showing attenuation of fibrosis following iron restriction or chelation further support the pathological importance of iron-driven macrophage activation in IPF (Pei et al., 2022; Yue et al., 2025).

Functionally, the immunometabolic state of macrophages in IPF drives several key pathological processes, including fibroblast activation, myofibroblast differentiation, and excessive ECM deposition, as well as epithelial cell injury and defective regeneration (Behmoaras et al., 2025). Unlike acute lung injury, where resolution is achievable, macrophage-mediated processes in IPF are self-sustaining and progressive, leading to irreversible structural remodeling of the lung (Xu et al., 2024). Importantly, these macrophages also contribute to the failure of normal repair mechanisms by disrupting epithelial-mesenchymal interactions and maintaining a pro-fibrotic microenvironment.

## 4 Therapeutic intervention through macrophage immunometabolism

### 4.1 Inhibition of glycolytic and FAO pathways

Targeting core metabolic circuits in macrophages has emerged as a rational strategy to recalibrate inflammatory and reparative programs in lung disease (Ogger and Byrne, 2021). Among these, glycolysis and FAO represent two dominant, and often opposing, bioenergetic axes that govern macrophage effector functions. Pharmacologic modulation of these pathways can reshape macrophage phenotype, cytokine output, and tissue interactions, offering opportunities to attenuate injurious inflammation or limit maladaptive repair, depending on disease context (Albers et al., 2025; Wang et al., 2024). Inhibition of glycolysis is primarily aimed at dampening hyperinflammatory macrophage responses that depend on rapid glucose flux. In activated pulmonary AMs, enhanced glucose uptake (e.g., via GLUT1) and upregulation of rate-limiting enzymes; hexokinase (HK), phosphofructokinase (PFK), and pyruvate kinase M2 (PKM2), support cytokine production and inflammasome activity (Ogger and Byrne, 2021; Lu S. et al., 2024; Woods et al., 2022). Targeting PFKFB3 (which regulates fructose-2,6-bisphosphate levels) suppresses glycolysis-driven inflammatory programs and mitigates neutrophil recruitment and vascular leakage (Liu C. et al., 2024). Mechanistically, glycolytic inhibition not only reduces ATP supply for biosynthesis but also alters immunoregulatory metabolite pools. Lower intracellular succinate levels can decrease stabilization of HIF-1α, thereby attenuating transcription of pro-inflammatory genes (Zhang and Lang, 2023). In parallel, reduced glycolytic flux diminishes NAD^+^/NADH imbalance and can indirectly modulate sirtuin activity and chromatin accessibility. However, the therapeutic window for glycolytic inhibition is narrow and context-dependent; excessive suppression may impair essential antimicrobial functions of macrophages, particularly in infectious settings such as bacterial pneumonia or tuberculosis. Thus, timing, dosing, and disease context are critical determinants of efficacy (Slama et al., 2024; Wang et al., 2026). In contrast, targeting FAO focuses on modulating macrophage programs associated with tissue repair, fibrosis, and long-term survival (Cui et al., 2025). FAO is regulated by key nodes including CPT1A, which controls mitochondrial import of long-chain fatty acids. Pharmacological inhibition of CPT1A (e.g., with etomoxir) reduces FAO-dependent oxidative metabolism and can limit profibrotic macrophage activity, particularly in conditions such as IPF where macrophages support fibroblast activation and ECM deposition (Al-Khami et al., 2017; Nandi et al., 2024; Huckestein et al., 2024). However, findings obtained with etomoxir, especially at the high concentrations commonly used in immunometabolic studies, require cautious interpretation because its immunomodulatory effects may arise from CPT1-independent depletion of intracellular coenzyme A rather than selective inhibition of FAO (Divakaruni et al., 2018). Accordingly, evidence supporting a specific role for CPT1A-dependent FAO should ideally be confirmed using low-dose pharmacological approaches, direct metabolic measurements, and genetic or orthogonal validation (Divakaruni et al., 2018). By constraining FAO, these interventions can decrease TGF-β production, myofibroblast differentiation, and collagen synthesis, thereby attenuating fibrogenic remodeling. Conversely, in highly inflammatory settings, selective enhancement of FAO, for example, via activation of peroxisome proliferator-activated receptors (PPARs), may promote anti-inflammatory macrophage phenotypes and facilitate resolution (Gu et al., 2026).

### 4.2 AMPK and mTOR modulators

The AMPK and mTOR pathways constitute a central regulatory axis that integrates nutrient sensing, cellular energy status, and inflammatory signaling in macrophages. In pulmonary macrophages, this axis serves as a critical determinant of immunometabolic state, managing the balance between catabolic and anabolic processes that underpin macrophage activation, plasticity, and function (Bao J. et al., 2025; Phair et al., 2023). AMPK acts as a cellular energy sensor that is activated in response to increased AMP/ATP ratios, hypoxia, and mitochondrial stress. Upon activation, AMPK promotes catabolic pathways, including FAO and autophagy, while suppressing energy-consuming anabolic processes (Yuan et al., 2020). In pulmonary macrophages, AMPK activation is strongly associated with anti-inflammatory and pro-resolving phenotypes, characterized by reduced NF-κB signaling, decreased production of pro-inflammatory cytokines (e.g., TNF-α, IL-1β, IL-6), and enhanced efferocytosis (Li Y. et al., 2025; Kaphalia et al., 2019). In addition, AMPK activation facilitates autophagic clearance of damaged organelles, including dysfunctional mitochondria, which is essential for maintaining macrophage metabolic fitness in inflammatory environments (Park et al., 2023; Zhang and Lin, 2016). Pharmacological activation of AMPK has demonstrated therapeutic potential in multiple pulmonary disease models. Agents such as metformin, AICAR, and other AMPK activators have been shown to attenuate macrophage-driven inflammation in lung injury by reducing inflammasome activation and cytokine release, while promoting epithelial barrier recovery (Rangarajan et al., 2018; Cheng et al., 2021; Idrovo et al., 2015). In COPD, AMPK activation may counteract cigarette smoke-induced mitochondrial dysfunction and impaired autophagy in AMs (He et al., 2025). In IPF, AMPK signaling has been implicated in limiting profibrotic macrophage activation by suppressing TGF-β-associated metabolic reprogramming and reducing fibroblast activation (Kheirollahi et al., 2019). These findings collectively support the concept that AMPK activation restores metabolic flexibility and promotes resolution-oriented macrophage functions. The mTOR pathway exists in two functionally distinct complexes; mTORC1, which drives biosynthetic and inflammatory programs, and mTORC2, which regulates cell survival, cytoskeletal organization, and aspects of macrophage polarization (Zhang H. et al., 2025). Pharmacological inhibition of mTOR, particularly through agents such as rapamycin and its analogues, has shown efficacy in modulating macrophage immunometabolism. mTOR inhibition suppresses glycolytic flux and inflammatory cytokine production, while simultaneously promoting autophagy and mitochondrial quality control (Huckestein et al., 2024). In IPF, suppression of mTOR-dependent anabolic metabolism can attenuate profibrotic macrophage activity and limit fibroblast activation (Huang et al., 2024). Despite their therapeutic promise, modulation of the AMPK-mTOR axis presents several challenges. The effects of these pathways are highly context-dependent, varying according to disease stage, microenvironmental conditions, and macrophage subset. For example, excessive suppression of mTOR signaling during early infection may impair host defense, whereas delayed inhibition may promote resolution of inflammation (Huynh et al., 2021). Similarly, while AMPK activation is generally protective, inappropriate activation in certain contexts may interfere with necessary immune responses (Wang et al., 2018). Furthermore, systemic targeting of these pathways can lead to off-target effects in other tissues, highlighting the need for lung-specific and macrophage-targeted delivery strategies.

### 4.3 Activation of antioxidant and NRF2 signaling pathways

In pulmonary macrophages, excessive production of ROS derived from mitochondrial electron transport chain dysfunction, NADPH oxidases, and inflammatory stimuli, acts as both a damaging agent and a signaling mediator that amplifies inflammatory cascades. Consequently, therapeutic strategies aimed at restoring redox balance have emerged as a critical avenue for modulating macrophage immunometabolism. Among these, activation of NRF2 pathway represents one of the most extensively studied and promising approaches. NRF2 is a key transcriptional regulator of antioxidant defense in pulmonary tissues, including AMs, where it drives cytoprotective gene expression through antioxidant response elements (AREs) (Mizumura et al., 2020). These target genes include key enzymes involved in glutathione synthesis (GCLC, GCLM), redox detoxification (NQO1, GPX, TXNRD1), and xenobiotic metabolism (Kang et al., 2021; Yang et al., 2019; Li W. et al., 2024; Sherwood et al., 2025). Activation of NRF2 therefore provides a coordinated response to oxidative stress, restoring intracellular redox homeostasis and protecting macrophages from oxidative damage. Importantly, NRF2 signaling is frequently dysregulated in COPD and IPF, where reduced NRF2 activity in AMs is associated with persistent oxidative stress and impaired immune function. Activation of NRF2 not only reduces ROS levels but also directly suppresses pro-inflammatory gene expression, including cytokines such as IL-1βand IL-6, through transcriptional interference mechanisms independent of redox control (Barnes, 2020; Li et al., 2022).

Furthermore, NRF2 also exerts profound effects on mitochondrial metabolism and bioenergetics, which are central to macrophage function. NRF2 activation enhances mitochondrial quality control, regulates oxidative phosphorylation, and limits excessive mitochondrial ROS production, thereby stabilizing cellular metabolism under stress conditions. By restoring mitochondrial integrity and metabolic flexibility, NRF2 activation helps re-establish functional macrophage responses, including phagocytosis and resolution of inflammation (Ryan et al., 2022; Kobayashi et al., 2016). Preclinical studies provide strong evidence supporting the therapeutic potential of NRF2 activation in lung disease. Pharmacological activators such as tert-butylhydroquinone (tBHQ), sulforaphane, and synthetic triterpenoids have been shown to attenuate lung injury, reduce inflammatory cytokine production, and improve survival in experimental models of acute lung injury (Veskemaa et al., 2021; Lu Q. et al., 2024; Harvey et al., 2011). These effects are mediated, at least in part, through reprogramming of macrophage polarization, with NRF2 activation promoting anti-inflammatory phenotypes and suppressing NF-κB-dependent inflammatory signaling. Liu et al. developed ROS-responsive liposomes encapsulating dimethyl fumarate (DTP@DMF) to enhance pulmonary NRF2 activation in IPF. In IL-4-stimulated macrophage-fibroblast cocultures and a bleomycin-induced mouse model, intratracheal DTP@DMF administration activated macrophage NRF2-HO-1 signaling, reduced ROS and TGF-β production, limited CD206^+^ macrophage accumulation, and attenuated myofibroblast differentiation and collagen deposition. Compared with free dimethyl fumarate, the liposomal formulation prolonged pulmonary retention and produced greater antifibrotic activity (Liu et al., 2022). Furthermore, Li et al. showed that GC-1 (sobetirome) activated NRF2 signaling in murine lung injury models and in mouse alveolar and human THP-1 macrophages, reducing mitochondrial ROS, inflammasome assembly, pyroptosis, and IL-1β/IL-18 release (Li B. et al., 2025). Similarly, in COPD models, restoration of NRF2 signaling has been associated with reduced oxidative stress, improved macrophage function, and enhanced bacterial clearance, highlighting its relevance in chronic disease settings. Harvey et al. found that activation of NRF2 signaling with sulforaphane restored defective bacterial phagocytosis and enhanced bacterial clearance by AMs in patients with COPD and cigarette smoke-exposed mice (Harvey et al., 2011). Emerging compounds targeting NRF2-KEAP1 interactions, including small-molecule inhibitors and protein-protein interaction modulators, have demonstrated the ability to restore NRF2 signaling in diseased macrophages and are currently under active investigation (Tran et al., 2019; Wang et al., 2023). Despite promising preclinical and *ex-vivo* findings, the clinical translation of NRF2 activators remains limited by inadequate pulmonary target engagement, compound-specific potency, and the potential off-target activity of electrophilic activators. In a multicenter phase II randomized, double-blind, placebo-controlled trial involving 89 patients with COPD, oral sulforaphane at 25 or 150 μmol/day for 4 weeks was systemically absorbed and well tolerated but failed to induce NRF2-regulated genes in AMs or bronchial epithelial cells and did not improve oxidative stress, inflammation, or pulmonary function (Barnes, 2020; Wise et al., 2016). These findings indicate that systemic exposure does not necessarily ensure sufficient NRF2 activation within diseased pulmonary macrophages. Furthermore, the variable potency and selectivity of different NRF2 activators emphasize the need for pharmacodynamic confirmation of lung-cell target engagement, optimized dosing, more selective KEAP1-NRF2 modulators, and macrophage-directed pulmonary delivery strategies (Barnes, 2020; Wise et al., 2016).

### 4.4 Targeting lipid signaling and iron homeostasis

Therapeutic manipulation of lipid signaling networks and iron handling represents a distinct arm of macrophage-directed immunometabolic intervention, with mechanisms that extend beyond canonical control of glycolysis or mitochondrial bioenergetics. In pulmonary macrophages, lipid-derived mediators and iron availability act as signal integrators that couple environmental stress to transcriptional programs governing inflammation, resolution, host defense, and tissue remodeling. Targeting these axes therefore enables reprogramming of macrophage function at the level of signaling and substrate utilization, rather than solely altering energy production (Nijmeh and Levy, 2021; Luo et al., 2025). Therapeutic strategies have shifted from global inhibition of lipid synthesis toward selective pathway modulation. For example, pharmacological blockade of leukotriene synthesis or receptor signaling can attenuate macrophage-driven amplification of inflammation, particularly in airway disease (Godson, 2020). However, greater specificity is achieved through pro-resolving approaches, including administration of resolvins, protectins, or maresins, which actively instruct macrophages to terminate inflammatory programs. These mediators enhance efferocytosis, suppress pro-inflammatory transcriptional activity, and promote a phenotype specialized for tissue repair (Serhan and Levy, 2025; Levy, 2012; Munir et al., 2019). Importantly, SPM-driven reprogramming is not immunosuppressive; rather, it restores macrophage capacity to resolve inflammation while preserving host defense (Yang et al., 2021).

Therapeutic targeting of lipid handling, through modulation of lipid uptake, intracellular trafficking, and cholesterol efflux, has been shown to restore macrophage competence. Activation of nuclear receptors such as PPARs and liver X receptors (LXRs) represents a key strategy in this context, as these transcription factors coordinate lipid metabolism with inflammatory gene expression. By promoting cholesterol efflux and normalizing lipid turnover, these interventions can reverse macrophage dysfunction and attenuate disease progression without broadly suppressing immune activity (Huang et al., 2019; Standiford et al., 2005; Carvalho et al., 2021; Yu et al., 2024). In parallel, excess intracellular iron promotes the formation of highly ROS through Fenton chemistry, thereby intensifying oxidative stress and perpetuating pro-inflammatory signaling. Accordingly, therapeutic strategies aimed at reducing iron burden, most notably through the use of iron chelators, have demonstrated efficacy in limiting oxidative injury and restoring macrophage homeostasis in preclinical lung disease models (Li Y. et al., 2024) (Table 3). By lowering labile iron pools, these agents reduce oxidative damage to cellular components and interrupt feed-forward inflammatory loops (Chen et al., 2022; Kim et al., 2023; Lovrić et al., 2022). An emerging area of interest is the intersection between iron metabolism and lipid peroxidation driven cell death pathways, particularly ferroptosis. In AMs, dysregulated iron handling and lipid accumulation can synergize to promote lipid peroxidation and cellular injury (Srinivasamurthy et al., 2025). Pharmacological inhibition of ferroptotic signaling, either by limiting iron availability or enhancing antioxidant defenses, offers a novel mechanism to preserve macrophage viability and function under conditions of oxidative stress (Srinivasamurthy et al., 2025). This approach is particularly relevant in diseases characterized by combined redox imbalance and lipid dysregulation.

### 4.5 Drug delivery strategies targeting pulmonary macrophages

Selective delivery of therapeutics to pulmonary macrophages has emerged as a pivotal strategy to translate immunometabolic interventions into clinically effective treatments. Given the central role of pulmonary macrophages in coordinating inflammatory, fibrotic, infectious, and tumor-associated processes, precise targeting of these cells enables modulation of disease-driving pathways while minimizing systemic toxicity (Morelli et al., 2025). This approach is particularly important for agents that affect metabolism or redox balance, where off-target effects in non-immune tissues may limit therapeutic applicability. Advances in nanotechnology, biomaterials, and inhalation-based delivery systems have therefore opened new avenues for cell-specific and lung-restricted modulation of macrophage function. One of the most promising approaches involves nanoparticle-based delivery systems, which can be engineered to exploit the intrinsic phagocytic capacity of AMs. Nanoparticles composed of lipids, polymers, or inorganic materials can be designed with specific size, charge, and surface characteristics that favor uptake by macrophages following inhalation or systemic administration (Jin et al., 2023; Iyer et al., 2015). For example, particles in the micron-to-nanoscale range with appropriate surface modifications are preferentially internalized by AMs via phagocytosis or endocytosis (Liu et al., 2025; Inoue et al., 2021). Surface functionalization with ligands targeting macrophage receptors, such as mannose receptors (CD206), scavenger receptors, or Fc receptors, further enhances specificity and uptake efficiency (Zlotnikov and Kudryashova, 2022; Zhou et al., 2018; Wendisch et al., 2021; van der Velden et al., 2024). These systems have been successfully used to deliver anti-inflammatory drugs, metabolic modulators, and nucleic acid based therapeutics directly to macrophages in preclinical lung disease models. Furthermore, inhalation-based delivery represents a particularly advantageous route for targeting lung macrophages, as it enables localized drug deposition within the respiratory tract and direct interaction with AMs at the air-liquid interface (Liu et al., 2022). Aerosolized formulations, including liposomes, polymeric nanoparticles, and dry powder inhalers, can achieve high local drug concentrations while reducing systemic exposure (Zhang H. H. et al., 2025; Craparo et al., 2016). Liposomal formulations, in particular, have shown strong translational potential due to their biocompatibility and ability to encapsulate both hydrophilic and hydrophobic compounds (Zhang et al., 2026). Clinically, liposomal delivery systems have already been explored for antimicrobial therapies in pulmonary infections, demonstrating enhanced drug accumulation within macrophages and improved therapeutic efficacy (Ferguson et al., 2023; Malinin et al., 2016). Beyond conventional small molecules, targeted delivery platforms have enabled the use of advanced therapeutic modalities, including RNA-based interventions and gene-editing technologies. siRNA, messenger RNA (mRNA), and CRISPR-based systems can be encapsulated within nanoparticles and delivered to pulmonary macrophages to modulate expression of key metabolic or inflammatory regulators (Mou et al., 2022; Elia et al., 2023). For example, silencing of genes involved in glycolysis, lipid metabolism, or inflammatory signaling has been shown to reprogram macrophage function (Wang et al., 2025). However, efficient and safe delivery of nucleic acids remains a major challenge, requiring optimization of carrier systems to ensure stability, cellular uptake, and endosomal escape. Despite significant progress, several challenges remain in the development of macrophage-targeted delivery systems. These include potential immunogenicity of carrier materials, variability in particle deposition within the lung, and clearance mechanisms that may limit therapeutic retention. In addition, disease-specific alterations in lung architecture, such as mucus hypersecretion, fibrosis, or tumor-associated changes, can affect drug distribution and uptake. Addressing these challenges will require continued refinement of delivery platforms, including the use of stimuli-responsive systems that release therapeutic cargo in response to local environmental cues such as pH, redox state, or enzymatic activity.

Collectively, the available evidence does not support a universal therapeutic strategy based on global activation or inhibition of a single metabolic pathway. The effects of targeting macrophage metabolism vary according to disease context, activation state, anatomical niche, and cellular ontogeny. For example, glycolytic inhibition suppresses the profibrotic phenotype of alveolar macrophages in experimental pulmonary fibrosis; however, glycolysis also contributes to macrophage antimicrobial activity, indicating that indiscriminate inhibition could compromise host defense (Zhang F. et al., 2020). Moreover, the reported requirement for FAO varies among macrophage activation states, and findings obtained using high-dose etomoxir may reflect CPT1-independent depletion of intracellular coenzyme A rather than selective inhibition of FAO (Van den Bossche and van der Windt, 2018). Future studies should therefore incorporate direct metabolic-flux measurements, genetic or orthogonal validation, and clear differentiation among resident alveolar, interstitial, and monocyte-derived macrophages. This distinction is particularly important because monocyte-derived and tissue-resident alveolar macrophages exhibit different contributions to pulmonary fibrosis. From a therapeutic perspective, temporally controlled and macrophage subset-selective correction of disease-associated metabolic dysfunction may be more effective than broad systemic modulation of immunometabolic pathways.

## 5 Conclusion

Immunometabolic remodeling is a central determinant of pulmonary macrophage function in chronic respiratory diseases. Altered metabolic pathways, redox balance, and mitochondrial activity critically regulate macrophage-driven inflammation, tissue remodeling, and fibrosis within the lung microenvironment. Increasing understanding of macrophage metabolic plasticity has identified pulmonary macrophage immunometabolism as a promising therapeutic target for chronic lung diseases.
